# Cumulative and Interactive Effects of Heavy Metal Mixtures Across Neurodegenerative Diseases: A Comparative Review of Alzheimer, Parkinson, Amyotrophic Lateral Sclerosis and Multiple Sclerosis

**DOI:** 10.3390/jox16050174

**Published:** 2026-09-16

**Authors:** Ivona Mitu, Ioana-Cezara Caba, Irina Macovei

**Affiliations:** 1 Department of Morpho-Functional Sciences II, Faculty of Medicine, Grigore T. Popa University of Medicine and Pharmacy, Universitătii Street, 700115 Iasi, Romania; ivona.mitu@umfiasi.ro; 2 Department of Pharmaceutical Sciences II, Faculty of Pharmacy, Grigore T. Popa University of Medicine and Pharmacy, Universitătii Street, 700115 Iasi, Romania; irina-macovei@umfiasi.ro; 3 Advanced Research and Development Center for Experimental Medicine “Prof. Ostin C. Mungiu”—CEMEX, Grigore T. Popa University of Medicine and Pharmacy, 16 Universitătii Street, 700115 Iasi, Romania

**Keywords:** heavy metal mixtures, neurodegeneration, Alzheimer’s disease, Parkinson’s disease, amyotrophic lateral sclerosis, multiple sclerosis, mitochondrial dysfunction, lead, cadmium, manganese

## Abstract

Heavy metals and metalloids are persistent environmental contaminants that accumulate in the central nervous system and interact with endogenous essential metals, yet most neurotoxicological research continues to treat metals as independent agents rather than as co-occurring mixtures. This review systematically compares the evidence for cumulative and interactive effects of heavy metal mixtures across four major neurodegenerative diseases—Alzheimer’s disease (AD), Parkinson’s disease (PD), amyotrophic lateral sclerosis (ALS), and multiple sclerosis (MS)—asking which metal combinations have been directly documented for each disease, and whether these patterns are disease-specific or shared. Following a PubMed search (January 2016–June 2026), 93 unique studies were included (AD: 49; PD: 38; ALS: 19; MS: 18; 31 shared across diseases). Combined exposure to lead, cadmium, arsenic, and mercury is linked to amyloid-beta accumulation and dementia risk in AD; manganese–vanadium co-exposure produces more severe dopaminergic damage in PD than either metal alone, with manganese activating the familial PD gene LRRK2; a multi-metal mixture in ALS was associated with a three-fold higher disease risk independent of genetic susceptibility; and MS showed almost no designed mixture studies despite considerable single-metal data, with conflicting findings across cohorts. Three general mechanisms were identified: competition at shared membrane transporters (notably DMT1), sequestration by metal-binding proteins, and direct synergistic or antagonistic interactions, in which essential elements can modulate toxic-metal handling. Mixture-statistics approaches (WQS, BKMR), already established for AD and PD, should be extended to MS to close this evidence gap.

## 1. Introduction

Heavy metals and metalloids are environmental contaminants that accumulate in biological tissues over a lifetime through combined dietary, occupational and environmental exposure routes [1]. A big concern is that they are not metabolized and instead they persist and interact with endogenous essential metals, accumulating preferentially in metabolically active tissues, including the central nervous system [2]. Metals relevant to human health are divided into two categories: (i) essential metals (e.g., iron (Fe), zinc (Zn), copper (Cu), selenium (Se), manganese (Mn)), which are considered cofactors for antioxidant enzymes, mitochondrial respiration and other physiological processes, and (ii) toxic metals and metalloids (e.g., lead (Pb), cadmium (Cd), mercury (Hg), arsenic (As)), which have no known biological function. Since both types of metals often share similar physicochemical properties, they compete for the same cellular entry points; therefore, toxic metal uptake can directly disrupt the handling of essential metals [3].

Within the central nervous system, this essential- and toxic-metal mishandling of the cell has been involved in four major neurodegenerative diseases that differ in their clinical presentation and, most importantly, in their neuronal target. Alzheimer’s disease (AD), the most common cause of dementia, is characterized by accumulation of amyloid-beta (Aβ) plaque and tau protein hyperphosphorylation, leading to progressive cognitive decline. Parkinson’s disease (PD) affects dopaminergic neurons of the substantia nigra, where alpha-synuclein aggregation drives motor symptoms like tremor, rigidity and bradykinesia. Patients with amyotrophic lateral sclerosis (ALS) present important muscle weakness, leading to paralysis, since this is a rapidly progressive motor neuron disease. Multiple sclerosis (MS), unlike the other three, is an autoimmune demyelinating disease of the central nervous system, producing multifocal lesions [4].

A substantial body of literature has linked individual metals to neurodegenerative disease risk, like Pb and Mn to Parkinsonian syndromes [5,6], Cd and As to AD [7], and mixed-metal dyshomeostasis to ALS and MS [8,9]. However, individuals are simultaneously exposed to complex mixtures through water, air, diet and occupational sources; therefore, real-world human exposure to a single metal never occurs in isolation. Statistical methods like weighted quantile sum regression (WQS) or Bayesian kernel machine regression (BKMR) have been used to show that combined metal exposures can produce effects that differ from those predicted by summing up the single-metal effects. Despite this, the majority of neurotoxicological research, including recent mechanistic reviews, continues to treat metals as independent agents.

This gap is only partially addressed in the literature. A recent review synthesized the combined neurotoxic effects of Pb, Cd and As through shared neural signaling pathways; however, it only focuses on these three metals and does not examine whether these effects differ across distinct neurodegenerative disorders [10]. Similarly, recent comprehensive reviews on trace element dyshomeostasis [11] and metallothionein biology in neurodegeneration [4] address essential versus toxic metal interactions, but largely at the level of single metals rather than a mixture, and do not perform an explicit cross-disease comparison. Therefore, our main goal is to systematically compare the evidence for metal-mixture effects across these four major neurodegenerative diseases (AD, PD, ALS and MS) asking two questions: (i) which metal combinations and interaction type (synergistic, antagonistic, additive) have been directly documented for each disease, and (ii) whether the pattern of interaction is disease-specific or shared across pathologies, potentially reflecting common underlying mitochondrial and redox mechanisms.

## 2. Literature Selection Methodology

Articles included in this review were selected through a structured search strategy to ensure adequate coverage and transparency. We searched the PubMed database on 1 July 2026 for papers published between 1 January 2016 and 30 June 2026 that responded to the following keyword structure: a metal/metalloid term + a mixture/co-exposure term + the disease. Zotero was used for article management.

The following four exact keyword strings were used:

- (“lead” OR “cadmium” OR “mercury” OR “arsenic” OR “manganese” OR “aluminum” OR “heavy metal”) AND (“metal mixture” OR “metal co-exposure” OR “combined metal exposure” OR “multi-metal” OR “co-exposure to metals”) AND (“Alzheimer’s disease” OR “AD”)
- (“lead” OR “cadmium” OR “mercury” OR “arsenic” OR “manganese” OR “aluminum” OR “heavy metal”) AND (“metal mixture” OR “metal co-exposure” OR “combined metal exposure” OR “multi-metal” OR “co-exposure to metals”) AND (“Parkinson’s disease” OR “PD”)
- (“lead” OR “cadmium” OR “mercury” OR “arsenic” OR “manganese” OR “aluminum” OR “heavy metal”) AND (“metal mixture” OR “metal co-exposure” OR “combined metal exposure” OR “multi-metal” OR “co-exposure to metals”) AND (“Amyotrophic lateral sclerosis” OR “ALS”)
- (“lead” OR “cadmium” OR “mercury” OR “arsenic” OR “manganese” OR “aluminum” OR “heavy metal”) AND (“metal mixture” OR “metal co-exposure” OR “combined metal exposure” OR “multi-metal” OR “co-exposure to metals”) AND (“Multiple Sclerosis”).

For multiple sclerosis, the abbreviation “MS” was excluded from the search string, since it frequently retrieved records related to metabolic syndrome, which falls outside the scope of this review.

The following inclusion criteria were considered: (i) articles reporting on exposure to two or more metals simultaneously, regardless of whether the analysis treated them jointly or individually, with an outcome relevant to neurotoxicity or neurodegeneration; (ii) reviews in which the toxicity of metals or metalloids constitute the central subject of discussion, which were retained for contextual framing and identification of research gaps; and (iii) English-language peer reviewed publications. Since several types of studies were included in the analysis, a classification scheme was used in the manuscript as follows: “Direct”—direct mixture/interaction evidence describing a formal mixture-statistics method (WQS, BKMR, quantile g-computation) or a designed multi-arm co-exposure experiment; “Multi-metal”—multi-metal measurement without formal interaction testing describing two or more metals measured in the same sample but analyzed individually; “Single-metal”—single-metal mechanistic or contextual evidence; and “Review”—protocols, narrative reviews or methodological papers. This classification is applied consistently across Table 1, Table 2, Table 3 and Table 4. Also, overall ratings in Table 5 reflect study design, sample size, exposure assessment, replication and mixture methodology, applied consistently across the four diseases.

The following exclusion criteria were considered: (i) studies addressing a single metal only; (ii) ecotoxicological studies with no human health endpoint; (iii) general reviews of neurodegenerative risk factors in which metals were mentioned only as an incidental example among a broader set of factors like pesticides, air pollution, etc.; and (iv) preprints without peer review.

**Figure 1:**
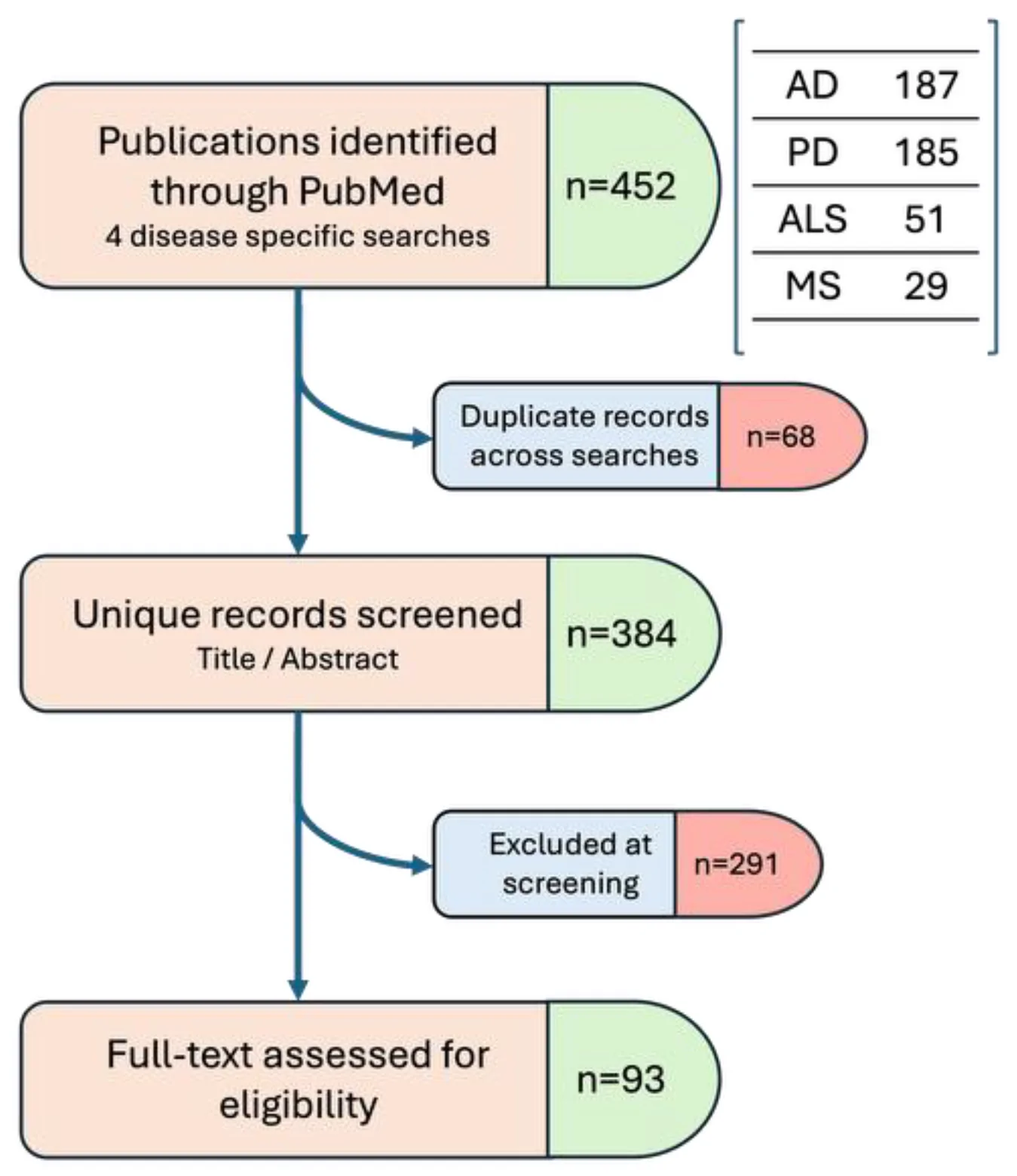
Publication selection workflow. AD = Alzheimer’s disease; PD = Parkinson’s disease; ALS = amyotrophic lateral sclerosis; MS = multiple sclerosis.

Figure 1 depicts the methodological workflow for the comparative study between the four target diseases. Duplicate records retrieved by more than one query were merged prior to screening in the Zotero software (version 9.0.6). Two reviewers screened together the title and abstract of the 384 articles identified in this stage and applied the inclusion and exclusion criteria. Disagreements arising during screening were resolved by a third reviewer. This step identified 291 articles that were excluded, yielding 93 unique studies included in the qualitative analysis: AD—49, PD—38, ALS—19, and MS—18, with 31 records shared across two or more disease-specific searches.

A supplementary non-systematic search was performed to include articles that document the mechanisms behind general metal–metal interactions. These are not restricted to the four target diseases and are well-established biochemical principles; therefore, the inclusion of papers for this general section is for contextual framing only.

The following terms regarding a possible interaction between metals will be used throughout this review: joint/mixture association—a relationship between an outcome and two or more simultaneously measured metals; synergism—a tested interaction in which the combined effect of metals exceeds the sum; antagonism—a tested interaction in which the combined effect is smaller than predicted by additivity; and additivity—a combined effect that matches the sum of individual effects.

## 3. General Mechanisms of Metal–Metal Interactions

### 3.1. Competition at Shared Membrane Transporters

The divalent metal transporter DMT1 mediates uptake not only of its preferred substrate, Fe, but also of other metals like Cd and Pb, and is expressed at every barrier a metal crosses on its route from ingestion to the brain. At the intestinal level, DMT1 is expressed in the apical membrane of enterocytes. Once the metal is absorbed, it is exported via the basolateral membrane into the bloodstream. Interestingly, in Caco-2 cell monolayers, Cd and Mn inhibited transport of Fe, while Pb and Zn did not, suggesting that Pb uses an independent hydrogen-driven mechanism to enter these cells [3]. DMT1 is also present in endosomes of brain capillary endothelial cells (the blood–brain barrier) [12] and in choroidal epithelial cells (blood–cerebrospinal fluid barrier) [13]. Critically, this transporter is itself inducible by metal exposure: Mn exposure upregulated DMT1 expression in choroidal epithelial cells in vitro, a mechanism proposed to produce a compartmental shift of Fe from blood to cerebrospinal fluid, indicating that exposure to one metal can increase the flux of others sharing the same transporter across the blood–cerebrospinal fluid barrier [13]. This transporter is not restricted to the cell membrane. DMT1 also mediates Fe and Mn uptake at the outer mitochondrial membrane, extending transporter-level competition to a level that is relevant to mitochondrial dysfunction [14].

### 3.2. Sequestration and Antioxidant Buffering

Se reduces Hg toxicity primarily through formation of biologically inert Hg-Se complexes with plasma selenoproteins [15]. An analogous antagonism has been reported for Cd: co-exposure with selenomethionine in human hepatic cells (HepG2) reduced the accumulation of Cd and enhanced cell viability, with Se supplementation increasing levels of selenometabolites and selenoprotein P [16].

At the protein level, metallothioneins constitute a further buffering system. In the isolated perfused rat liver, Cd completely displaced Zn from normal levels of metallothionein and on a one-to-one basis from elevated levels, both in vivo and ex vivo, whereas Hg displaced Zn from the same protein less efficiently in vivo [17]. In Cu-preinduced liver, where metallothionein contains both Zn and Cu, Cd displaced Zn selectively without affecting Cu levels; Hg, despite having the highest in vitro affinity for the protein among the metals tested, did not displace Cu as predicted, but instead increased Cu retention in metallothionein alongside its own incorporation [18]. These findings suggest that a physiological Zn- and Cu-regulatory protein can be selectively co-opted by a toxic metal, with the specific pattern of displacement depending on which essential metals are already bound rather than on in vitro affinity alone.

### 3.3. Direct Synergistic and Antagonistic Interactions

A plasmid DNA assay directly comparing single-metal and combined exposure found that binary mixtures revealed synergistic effects between Cd and Hg, most pronounced at a 1:1 molar ratio, while Zn or Se exhibited antagonistic effects with Hg or Cd. Moreover, the majority of multi-metal combinations in this study reduced DNA damage compared to individual metals [19]. Mice exposed simultaneously to Pb, Hg, and Cd through drinking water for 28 days showed worse motor and cognitive performance than mice exposed to any single one of these metals, and striatal dopamine levels dropped further under the combined exposure than under any single-metal condition [20]. These findings support the idea that combined metal exposure does not simply sum single-metal effects.

## 4. Disease-by-Disease Comparative Profile

The general biochemical mechanisms through which metals interact with one another and further generate a cascade of cellular consequences have important implications, particularly in neurodegenerative diseases. The depth and type of available evidence differ substantially across these conditions, ranging from large prospective cohorts applying formal mixture-statistics methods to case reports and single-mechanism cell culture studies. All four neurodegenerative disorders discussed in this review share common ground regarding mechanisms of biochemical dysfunction. Nevertheless, studies focused on a specific disease often discuss different metal combinations and may yield distinct findings.

### 4.1. Alzheimer’s Disease

Population-level evidence indicates that metal mixtures carry more explanatory weight for cognitive decline and dementia risk than any single metal alone. In the Multi-Ethnic Study of Atherosclerosis (MESA), a prospective cohort of over 6300 participants, individuals in the 95th percentile of a nine-metal urinary mixture had a 71% higher risk of incident dementia than those in the 25th percentile [21]. A metallomics study of 514 older adults in Beijing found that a Cu-Pb mixture was negatively associated with cognitive scores, and that Se included in the same mixture attenuated this neurotoxicity, with twenty cognition-related genes (including APOE and APP) showing opposite regulatory responses to Se versus Cu/Pb exposure [22]. Moreover, among Chinese older adults, higher Se values weakened the negative association between combined As-Cd-Pb exposure or exposure to Pb alone and cognitive performance [23], and in American older adults, Se was associated with better cognitive function, while Cd and Pb predicted worse performance [24]. A cross-sectional study of aluminum (Al)-plant workers found that an aluminum–lead–lithium mixture reduced cognitive scores synergistically, with Zn showing an independent protective association [25]. Coal miners exposed to a sixteen-metal urinary mixture showed cognitive decline, together with low levels of a proposed candidate biomarker, sTREM2 [26]. Since women are known to be more prone to AD and related dementia as they get older, a systematic review focusing on a sex-specific approach identified 34 epidemiological studies over an 11-year window, reporting adverse associations for Cu, Cd, As, Pb, and Mn alongside protective associations for Se, Fe, and Zn [27]. Nevertheless, the same review also found studies with no association reported. These findings altogether highlight the potential protective role of Se regarding AD or related dementias in elder subjects and highlights the detrimental effect of mixtures on cognitive function.

Direct experimental evidence reinforces these associations. In an attempt to establish a cost-effective ex vivo model for studying AD, Korde and Humpel [28] found that Al, Pb, and Cd together potentiated amyloid-beta plaque-like pathology in organotypic mouse brain slices beyond what pharmacological modulators alone achieved. Mechanistically, Pb exposure increased mitochondrial translocation of the Cu transporter COX17 in APP/PS1 mice and Aβ-treated microglia, producing mitochondrial Cu overload and therefore damage to the mitochondria and microglial activation. This process leads to neuroinflammation, aggravating Alzheimer-like pathology [29], and represents a good argument for how a toxic metal disrupts an essential metal’s mitochondrial handling. An in silico toxicogenomic analysis of Pb, As, gold (Au), Cu, Fe, and Al found that mixed exposure altered apoptosis- and inflammation-related genes converging on PI3K-Akt, p53, and NF-κB signaling, and identified sixteen microRNAs implicated in cognitive impairment, together with five possible target genes—BAX, CASP3, BCL2, TNF, and IL-1B—for future neuroprotective treatments [30]. One study outside of the systematic search window stands as a valid argument and a possible foundational experiment establishing the same relevant As-Cd-Pb mixture dosing in this disease context. Administration of As-Cd-Pb together to young rats at environmentally relevant doses reported dose-dependent Aβ accumulation [7].

Neuroimaging and tissue-level evidence converge on the same conclusion. In welders exposed to a metal mixture, diffusion tensor imaging revealed augmented diffusivity in the medial temporal lobe and reduced fractional anisotropy in entorhinal and parahippocampal cortices, alongside poorer cognitive performance [31], with a companion study extending this finding to the basal ganglia [32]. Postmortem comparison of brain tissue and ventricular fluid found that fluid metal levels did not represent brain tissue concentrations, with brain Fe elevated and As/Cd reduced in Alzheimer’s cases relative to controls [33]. In cerebrospinal fluid (CSF), heavy, essential and non-essential metal concentrations correlated positively with phosphorylated tau isoforms in CSF, neurofilament light chain, and other Alzheimer biomarkers [34]. At the cellular level, Se reduces Cd-induced cytotoxicity in SH-SY5Y neurons via upregulation of thioredoxin reductase 1, strengthening antioxidant capacity of the cell [35].

Reviews and meta-analyses situate these findings within broader frameworks. A review of As, Mn, Pb, and Cd in AD converges on oxidative stress, mitochondrial dysfunction, and disrupted autophagy as shared pathways [36]. The autophagy–lysosomal pathway is centrally relevant because it is the system responsible for clearing amyloid precursor protein and its Aβ metabolites, and heavy metals disrupt this clearance directly at the metal-specific step: Pb reprograms amyloid precursor protein (APP) transcription, Hg suppresses the Aβ-degrading enzyme neprilysin, and Cd and Mn impair Aβ degradation itself. Therefore, Aβ accumulates not only because more is produced, but also because the mechanism meant to remove it is disabled by the same metals driving its production. This mechanism does not operate independently of aging; therefore, metal-induced and age-related autophagic efficiency decline are thought to act synergistically, rather than additively [37]. Another recent paper addresses the role of metals on epigenetic alterations and reports how Cd, Fe, As and lithium (Li) disrupt DNA methylation through distinct upstream routes: Cd via TET/DNMT dysregulation, Fe via DMT1-mediated oxidative stress, As via direct DNMT inhibition and Li via GSK-3β pathway. Most of these mechanisms lead to the same downstream signature: hypomethylation of APP, PSEN1, PSEN2, and GSK-3β, alongside hypermethylation of ANK1, RPL13, RHBDF2, DUSP22, and SORL1, enhancing high Aβ and tau phosphorylation and aggregation in AD [38].

Taken together, few of the findings above report an explicit interaction like synergism (e.g., between Al, Pb and Li on cognitive scores [25]) or antagonism (e.g., Se attenuates Cu-Pb [22] and As-Cd-Pb [23] toxicity). The concept of synergism is also mentioned between metal-induced and age-related autophagic decline [37]; however, it is more of a proposed mechanism rather than a statistically proven result. The remaining mixture findings, despite several relying on formal mixture-statistics methods (WQS, BKMR, qgcomp), report joint or combined associations without explicitly testing whether the effect departs from additivity.

Table 1 summarizes the studies included in this review addressing metal exposure in relation to Alzheimer’s disease, grouped by shared metals, methodology and convergent findings.

**Table 1:** Synthesis of studies on metal exposure and Alzheimer’s disease.

Metals/Mixture Studied	Design/Method	Key Findings (Synthesized)
Pb, Cd, Hg, Mn, Se, Cu, As, Co, W, U, Zn, Ba, Cs, Tl, Al, Li (varies by study)	[Direct] 7 independent human cohorts; 6 use formal mixture statistics (WQS, BKMR, qgcomp) on blood/urine metal panels and 1 uses logistic regression	Metal mixtures—not single metals—consistently predicted worse cognition or higher dementia/MCI risk, dose-dependently; two of the seven studies specifically found that Se attenuated the toxic-metal mixture effect (Cu-Pb-Se; Pb-Cd-Hg-Mn-Se) [21,22,25,26,39,40,41]
Al, Pb, Cd, Fe, Cu	[Direct] 4 experimental models (organotypic brain slices; APP/PS1 mice + BV-2 cells; two rat models) exposed to defined metal combinations	Combined metal exposure produced greater Aβ/tau pathology than any single metal in every model tested; two studies identified a specific, reversible mechanism: mitochondrial Cu overload via COX17 (Pb + Cu), and antioxidant rescue by spermine or berberine (Al + Fe; Al + Cd + fluoride) [28,29,42,43]
Pb, Cd, As, Hg/MeHg, Au, Cu, Fe, Al	[Direct] 4 in silico toxicogenomic analyses (Comparative Toxicogenomics Database gene-network mining)	All four independent analyses, despite testing different metal combinations, converged on the same core apoptosis/oxidative-stress gene set (BAX, CASP3, BCL2, TNF) as the shared molecular signature of metal-mixture neurotoxicity; one analysis extended this convergence to ALS and PD, identifying SOD2 as a gene mutual to all three diseases [30,44,45,46]
Se, Zn vs. Cd	[Direct] 2 in vitro studies, SH-SY5Y neuronal cells	Both Se and Zn, tested independently, attenuated Cd-induced cytotoxicity via antioxidant mechanisms—direct cellular-level confirmation of essential-metal protection against a toxic metal, though Se’s protection did not extend to differentiated cholinergic cells in one study [35,47]
Mn, V, Fe, Cu (welding-fume mixture)	[Direct] 2 companion case-control studies in the same welder cohort, diffusion tensor/T1 MRI	Mixed occupational metal exposure altered MRI metrics in both the medial temporal lobe and basal ganglia; Mn and V showed effects individually and jointly as a mixture [31,32]
As, Cd, Hg, Ni, Pb, Tl + essential metals (CSF); Fe, Cu, Zn, As, Cd, Mn (postmortem/tissue)	[Multi-measured] CSF/plasma biomarker correlation (*n* = 193); postmortem brain-vs-fluid comparison; single-neuron synchrotron imaging (*n* = 7)	Heavy and essential metal levels correlated with CSF markers of AD pathology in living patients, but postmortem data showed brain tissue and ventricular fluid levels are not interchangeable, and single-neuron imaging revealed substantial cell-to-cell variation in metal accumulation—together cautioning against treating any single fluid compartment as a reliable proxy for brain metal burden [33,34,48]
Cd, Pb, Hg, As, Se	[Multi-measured] Case-control study (*n* = 434, propensity-matched) and systematic review/meta-analysis (22 studies, *n* = 3346)	The case-control study linked As metabolite profile and low Se to elevated AD risk; the meta-analysis, testing four metals across a much larger literature, found only Cd significantly elevated in AD patients versus controls, with Pb, As, and Hg not significantly different [49,50]
Cd, Pb (+As, Mn, Hg)	[Multi-measured] Ecological, US geographic correlation (topsoil, sewage sludge, well water, infant blood)	Cd and Pb in sewage sludge, used as an environmental exposure proxy, were significantly associated with neurodegenerative disease prevalence across the United States [51]
Pb, As, MeHg	[Multi-measured] In vitro hippocampal cell proteomics	Established a relative potency ranking (Pb < As < MeHg) while identifying mitochondrial dysfunction and oxidative stress as pathways shared across all three metals [52]
Pb, Hg, Cd, Mn, As, Cu (+Mg in one)	[Review] 4 narrative reviews, autophagy–lysosomal/mitochondrial mechanism focus	All four reviews converge on the same conclusion: heavy metals impair the autophagy–lysosomal pathway and mitochondrial function (electron transport chain, mtDNA integrity), proposed as a mechanism shared between AD and PD [37,53,54,55]
As, Mn, Hg, Al, Pb, Ni, Cd, Cu, Zn, Fe, Co (varies)	[Review] 4 narrative reviews, each centered on a distinct specific mechanism or therapeutic angle	Four reviews each foreground a different, less conventional angle: astrocyte-mediated metal accumulation and iron-induced astrogliopathy, exosomal miRNA transport linking peripheral metal exposure to brain neuroinflammation, ferroptosis as a shared metal-induced cell-death pathway, and nanoparticle-based chelation as a therapeutic strategy; these do not converge on one shared finding but each adds a distinct mechanistic candidate [56,57,58,59]
Pb, Al, Hg, Mn, Cd, As (recurring core set)	[Review] 9 narrative reviews, general multi-metal mechanistic overviews	Substantial overlap across all nine: the same core set of metals (Pb, Al, Hg, Mn, Cd, As) is repeatedly implicated via oxidative stress, neuroinflammation, and BBB disruption; individual reviews add specific detail without contradicting this shared core: Pb-BBB/epigenetic effects, Cd-p53/p21/Rb senescence, As-nitric oxide signaling, Mn-glutamate excitotoxicity, and overlap with PD mechanisms [36,44,45,60,61,62,63,64,65]
Cu, Cd, As, Pb, Mn (adverse); Se, Fe, Zn, Al, Si (protective or mixed)	[Review] 2 broad reviews synthesizing epidemiological studies (34 studies; 60 of 4784 screened)	Both reviews report the same overall pattern: findings across the underlying literature are inconsistent and metal-specific, with adverse associations most consistent for Cu/Cd/As/Pb/Mn, and protective or mixed evidence for Se/Fe/Zn; moderate evidence also implicates Al in general dementia risk [27,66]
Cd, Fe, As, Cu, Li	[Review] 1 narrative review, epigenetics-specific	Proposes that these five metals converge on the same downstream DNA methylation signature at AD-relevant genes despite acting through distinct upstream mechanisms [38]
Mn, Zn, Fe, Cu, Ni	[Review] 1 narrative review, aging-specific	Concludes that age-related metal accumulation compounds mitochondrial dysfunction and calcium dyshomeostasis already present in aging neurons [67]
18 metals (evidence-map protocol); Mn (methods chapter)	[Review] 1 published protocol; 1 laboratory methods chapter	No primary findings in either—cited as methodological/procedural context only, not as evidence [68,69]

WQS = weighted quantile sum regression; BKMR = Bayesian kernel machine regression; MCI = mild cognitive impairment; MRI = magnetic resonance imaging; Aβ = amyloid-beta; CSF = cerebrospinal fluid; BBB = blood-brain barrier; [Direct] = formal mixture statistics or a designed multi-metal co-exposure, [Multi-measured] = two or more metals measured/compared but analyzed individually, [Single-metal] = one metal only, [Review] = narrative review, protocol or methodological paper.

### 4.2. Parkinson’s Disease

Two studies from the same group examined Mn-vanadium (V) co-exposure in Parkinsonian models. Ngwa et al. [70] found that combined intranasal Mn-V exposure in an animal model produced more severe olfactory and nigral damage than either metal alone, with an important decrease in tyrosine hydroxylase and dopamine. Moreover, they reported high levels of 4-hydroxynonenal (an important marker for oxidative stress) in the striatum and substantia nigra [70]. A follow-up study in transgenic mice expressing human alpha-synuclein A53T found that the same Mn-V mixture produced significant motor and olfactory deficits specifically in the transgenic animals, while wild-type mice given the identical mixture were largely unaffected [71]. This second study is evidence of a gene–environment interaction and lacks single-metal comparison arms; therefore, it cannot independently confirm metal–metal synergy on its own. However, these studies provide evidence that a metal mixture interacts with, rather than simply adds to, genetic Parkinsonian susceptibility. At a cellular level, Zn produced the most severe dopaminergic loss, DNA damage, and mitochondrial dysfunction among Mn, Zn, and Cu chlorides tested against the classic Parkinsonian toxin 6-hydroxydopamine in SH-SY5Y cells [72].

A recent analysis linked oxidative stress to mixed-metal exposure and Parkinsonian risk in humans. This case-control study of urinary trace elements highlighted an elevated PD risk in subjects with both increased exposure to Mn and Pb, and insufficient intake of Se, Cd, chromium (Cr) and nickel (Ni). Moreover, oxidative stress levels (8-hydroxy-2-deoxyguanosine [8-OHdG]) mediated approximately 11.6% of the combined Mn-Pb effect. Interestingly, BKMR models showed an increase in the risk for PD both in deficiency and in excess of metals, strengthening the need to better characterize the link between PD and metal exposure [73]. A comprehensive review of Fe, Hg, Mn, Cu, and Pb in PD explicitly notes that interactions among mixture components may produce synergistic toxicity beyond single-metal effects [74].

Mechanistically, Mn exposure robustly activates LRRK2, a kinase whose mutations are associated with familial and sporadic PD, through a redox-dependent pathway involving impaired mitochondrial respiration and increased reactive oxygen species [75]. This study falls outside our structured search window but is retained as foundational mechanistic evidence not presented elsewhere. Complementary structural studies have examined cobalt (Co) and Mn binding to alpha-synuclein directly [76], and cell-based work has shown that As and Cd jointly influence alpha-synuclein aggregation [77]. Comparative reviews document overlapping Fe, Cu, Zn, and Mn dyshomeostasis across AD and PD [45], and broader reviews addressing occupational exposure, physiological/pathological mechanisms, and hair-based metal dysregulation in PD converge on oxidative stress, mitochondrial dysfunction, and neuroinflammation as shared downstream pathways [78,79,80]. In particular, hair metal profiling in PD subjects revealed a coordinated multi-element signature: low levels of Fe and Cu, alongside high values for Mn and As. In a parallel MPTP mouse model, the Fe deficiency was linked to gut dysbiosis, characterized by upregulated bacterial iron-acquisition genes and downregulated iron-transport genes (including DMT1). These findings suggest that intestinal microbiota may compete with the host for Fe in PD [80].

The majority of the mixture findings described above present joint or combined effects, like oxidative-stress mediation and joint influence on protein aggregation, without formally testing or labeling them as synergistic [73,77]. Mn-V co-exposure is the most important finding that involves a directly tested metal–metal synergism [70].

Table 2 summarizes the studies included in this review addressing metal exposure in relation to PD, following the same grouping structure as Table 1.

**Table 2:** Synthesis of studies on metal exposure and Parkinson’s disease.

Metals/Mixture Studied	Design/Method	Key Findings (Synthesized)
Pb, Al, Hg, Mn, Cd, As (recurring core) + Fe, Cu, Zn, Ni in some	[Review] 13 items: 12 narrative reviews + 1 single-neuron synchrotron imaging study	Overlap with the AD literature: oxidative stress, mitochondrial dysfunction, BBB disruption, and autophagy impairment recur as shared mechanisms across the same core metal set; the imaging study adds single-neuron-resolution evidence that individual locus coeruleus neurons vary in toxic/essential metal content [37,45,48,51,54,55,59,60,62,64,65,67,68]
Se, Zn vs. Cd	[Direct] 2 in vitro studies, SH-SY5Y neuronal cells	Overlap with the AD literature: direct cellular confirmation of essential-metal protection against a toxic metal [35,47]
As, Mn, Hg, Al, Pb, Ni, Cd, Cu, Zn, Fe (astrocytes); As, Co, Cd, Fe, Mg, Mn, Ni, Hg, Zn, Se (ferroptosis)	[Review] 2 narrative reviews, each proposing a distinct specific cell-biology mechanism	Overlap with the AD literature: astrocyte-mediated metal accumulation and iron-induced astrogliopathy; ferroptosis (Fe-dependent cell death via GPX4/Xc-failure, with Se being protective)—the two mechanisms do not converge on a shared finding [56,58]
Pb, As, MeHg, Cd	[Multi-measured] In vitro hippocampal proteomics; in silico toxicogenomic gene-network mining; narrative mechanism-of-action review	Three independent approaches converge on shared binding targets for this quaternary mixture (NMDA receptor, Na^+^-K^+^ ATPase, Ca^2+^ signaling, glutamate transmission) and shared downstream pathways (mitochondrial dysfunction, oxidative stress, SOD2); one study established a relative potency ranking of Pb < As < MeHg [52,81,82]
Co, Ni, Hg, Cr, Tl; Cu, As, Cd, Fe, Li; Hg, Pb, Cu, Zn, Fe, Mn, Al, As, Cd, Se; Fe, Hg, Mn, Cu, Pb	[Review] 4 narrative reviews specifically on metals and PD mechanisms	All four consistently implicate oxidative stress, mitochondrial dysfunction, and alpha-synuclein aggregation as shared downstream consequences of metal exposure in PD, though each emphasizes a different subset of metals; one explicitly notes that interactions among mixture components may produce synergistic toxicity beyond single-metal effects [5,79,83,84]
Mn, V	[Direct] 2 independent mouse studies (same research group), intranasal co-exposure	Ngwa et al. [70]: 4-arm design (control/Mn/V/Mn + V)—genuine metal–metal synergy, co-treatment produced most severe deficits. Kanthasamy et al. [71]: same mixture in WT vs. A53T transgenic mice—genuine gene-environment interaction, but no single-metal arms, so it cannot independently confirm metal–metal synergy.
Mn, Zn, Cu	[Multi-measured] In vitro, SH-SY5Y cells, benchmarked against the classic PD toxin 6-OHDA	Zn was the most potent dopaminergic toxin of the three metals tested; Mn and Cu at LC50 produced a response similar to 6-OHDA, suggesting distinct upstream mechanisms converging on a common idiopathic PD-like phenotype [72]
As, Cd; Co, Mn	[Direct] 2 in vitro biophysical/structural studies (aggregation kinetics; native mass spectrometry)	Both metal pairs directly bind and alter alpha-synuclein aggregation: As and Cd become incorporated into amyloid fibers and accelerate nucleation while reducing aggregate clearance in yeast cells; Co and Mn bind the C-terminal region and induce structural compaction of the protein [76,77]
Mn, Pb, Cr, Ni, Se, Cd (urinary); Ba, Cd, Co, Cs, Mo, Pb, Sb, Tl, U (NHANES)	[Direct] 2 independent human cohort/case-control studies using formal mixture statistics (BKMR, WQS, quantile g-computation)	Metal mixtures elevate PD risk beyond single-metal effects, though the dominant contributors differ by study (Mn 73.7% + Pb 9.3% in one; Mo + Co dominant in the NHANES WQS model) [73,85]
Fe, Cu (low); Mn, As (high); Zn (unchanged)	[Multi-measured] Cross-sectional human hair analysis + MPTP-induced mouse model	Lower hair iron (Fe) and copper (Cu) alongside higher manganese (Mn) and arsenic (As) distinguished PD patients from controls; the mouse model linked the iron (Fe) deficit specifically to gut microbiota dysbiosis and impaired intestinal iron (Fe)-transport gene expression (DMT1, FPN), proposing a gut–brain axis mechanism [80]
Pb, Cd; Fe, Mn	[Single-metal] Rat model (Pb, Cd, N-acetylcysteine intervention) and SH-SY5Y cells (Fe, +Mn, butyrate, butyrate + nicotine intervention)	Both studies found combined toxic-metal exposure upregulated PD-related genes or toxicity markers (Parkin, Pink1, LRRK2, SNCA for Pb, +Cd), and both identified an antioxidant-based intervention that protected against the combined-metal damage, though via distinct mechanisms [86,87]

Rows highlighted in light orange represent studies shared with the other neurodegenerative diseases covered in this review. BBB = blood–brain barrier; LC50 = lethal concentration 50; 6-OHDA = 6-hydroxidopamine; ROS = reactive oxygen species; LRRK2 = leucine-rich repeat kinase 2; NHANES = National Health and Nutrition Examination Survey; WQS = weighted quantile sum regression; BKMR = Bayesian kernel machine regression; MPTP = 1-methyl-4-phenyl-1,2,3,6-tetrahydropyridine; DMT-1 = divalent metal transporter 1; FPN = ferroportin; [Direct] = formal mixture statistics or a designed multi-metal co-exposure, [Multi-measured] = two or more metals measured/compared but analyzed individually, [Single-metal] = one metal only, [Review] = narrative review, protocol or methodological paper.

### 4.3. Amyotrophic Lateral Sclerosis

The strongest quantitative mixture evidence identified across all four diseases concerns ALS. In a Michigan case-control cohort, a combined environmental risk score across a multi-metal panel, with Cu, Se, and Zn being individually significant, was associated with an approximately three-fold increase in ALS risk and reduced survival, independent of each participant’s polygenic risk score for either ALS or metal handling [8]. This provides direct evidence that mixture-level environmental exposure and genetic susceptibility act at least partly independently in ALS. Evidence from cerebrospinal fluid is more equivocal: in a case-control study measuring Pb, Cd, and Hg directly in CSF from 38 ALS patients and 38 controls, ALS patients showed higher median Pb but lower Cd and Hg, with no significant dose–response relationship for any of the three metals [88]. This study confirms that not every metal combination tested matters for ALS, even though the broader multi-metal mixture literature shows a robust overall association. A pilot urinary case-control study found significantly higher Pb and Cu, and a subtle Mn increase, in ALS patients who had not changed residence since diagnosis [89].

Mechanistically, heavy metal neurotoxins have been shown to induce TDP-43 accumulation, the defining pathological protein of ALS [90]. Mn-Cu co-exposure in a yeast model produced characteristic histone H3 post-translational modification changes, proposed as an epigenetic mechanism potentially shared between ALS and frontotemporal dementia [91]. Genotoxic profiling has distinguished metal-induced DNA damage patterns between sporadic and familial ALS [92], and DNA methylation variability has been linked to self-reported heavy metal exposure in an ALS cohort [93]. Interestingly, a specific pattern of metal exposure could help distinguish familial from sporadic ALS: Co, Cu and Zn show strong associations with ALS-linked genetic mutations, while As, Al and uranium (U) do not [92]. Ecologically, regional ALS incidence in Ferrara, Italy, has been associated with ambient air metal pollutant levels, suggesting that Cu may have a role in ALS developing in the general population [94]. An ALS case cluster has been documented among automobile workers with combined occupational exposure to heavy metals, organic solvents, and diesel exhaust, concluding that this exposure was significantly supported by the scientific evidence as a contributing cause of their disease [95].

No finding in this section involved a formally tested metal–metal synergism or antagonism. The strongest evidence is the one reported by the multi-metal environmental risk score [6], reporting a joint, dose-dependent association independent of genetic risk, but without testing whether individual metals interact with one another.

Table 3 summarizes the studies included in this review addressing metal exposure in relation to ALS disease.

**Table 3:** Synthesis of studies on metal exposure and amyotrophic lateral sclerosis.

Metals/Mixture Studied	Design/Method	Key Findings (Synthesized)
Pb, Al, Hg, Mn, Cd, As (recurring core) + other metals	[Review] 7 items: narrative reviews, 1 protocol, 1 in silico toxicogenomic analysis, 1 ecological study	Overlap with AD and PD: same recurring core mechanisms (oxidative stress, mitochondrial dysfunction, BBB disruption) across the same core metal set [51,56,59,61,64,68,81]
Multi-metal panel in plasma and urine (individual metals significantly associated with risk/survival: copper, selenium, zinc)	[Direct] Case-control study (*n* = 454 ALS/294 controls), ICP-MS on plasma and urine; ALS and metal polygenic risk scores computed from independent GWAS/literature-selected SNPs	Elevated Cu, Se, and Zn were individually linked to ALS risk and survival, and a combined environmental risk score across all measured metals showed a strong, dose-dependent association with both greater risk (OR ~3) and worse survival (HR ~1.4)—independent of genetic risk, and correlated with known occupational and non-occupational exposure sources [8]
Mg, Cu, Se, Fe, Mn, V, Zn, Al, As, Co, Ni, Hg, Pb, Cd, Pd (15 elements); Pb, Cd, Hg	[Multi-measured] 2 case-control/observational studies measuring CSF metal levels directly (ICP-MS)	Mixed evidence. One study found Se and As elevated above reference values in ALS patients, with Cu, Fe, Mn, Zn, Al, Ni, and Pb differing between bulbar and spinal onset subtypes [96]. A methodologically distinct case-control study found higher Pb in ALS, but lower Cd and Hg than controls, with no significant dose–response relationship for any of the three metals—the authors concluded their data did not support a role for these metals in ALS etiology [88]. This null/mixed result should temper strong causal claims about this specific three-metal combination.
Hg, Ag, Bi (autometallography)	[Multi-measured] Histological study, spinal cord tissue from 50 individuals without motor neuron disease	Heavy metals detected in spinal interneurons in 33% of individuals aged 61–95, absent at younger ages, suggesting age-related accumulation could predispose inhibitory interneurons to damage relevant to ALS pathogenesis. Note: this study used non-ALS control tissue, not ALS patients directly [97].
Pb, Mn, Se, Cu, Zn (urinary); Pb, Cd, Al, Hg, Mn, Fe, Cu, Zn, Se, Mg, Ca (blood/urine/hair)	[Multi-measured] 2 case-control studies (*n* = 42 and severity-stratified cohort)	Both identify Pb as a consistent risk-associated metal (elevated urinary Pb in one; Pb as risk factor in blood in the other), while Se showed a protective association in one study; a subtle Mn increase and elevated Cu also appeared in the urinary study [89,98]
As, Cd, Pb, Hg, Cr	[Review] Umbrella review of 35 meta-analyses, 103 health outcomes (AMSTAR2-graded)	Pb showed a significant association specifically with ALS (equivalent OR 1.46, 95% CI 1.16–1.83, credibility class III/suggestive) among the 103 health outcomes evaluated for these 5 metals—the only ALS-specific finding within a much broader multi-disease evidence synthesis [99]
Pb, Hg, Sn	[Multi-measured] In vitro (cultured cells) + in vivo (mouse cortex) mechanistic study	Pb and meHg directly disrupted TDP-43 homeostasis, triggering nuclear granule accumulation and increased splicing activity; Pb specifically promoted dose-dependent phase separation of TDP-43 in vitro—the clearest direct mechanistic link identified between a specific metal and the primary pathological protein of ALS [90]
Heavy metals + organic solvents + diesel exhaust; Ag, Al, Cd, Cr, Cu, Fe, Mn, Pb, Se (ecological)	[Multi-measured] Formal occupational case evaluation (*n* = 3 workers) + ecological correlation study (*n* = 62 ALS cases, moss/lichen biomonitoring)	A Korean government committee attributed ALS in three automobile workers to combined 15–33-year occupational exposure to heavy metals (primarily Pb from engine work), organic solvents, and diesel exhaust. Separately, an ecological study in Italy found ALS case density correlated most strongly with Cu air pollution specifically—not with the other 8 metals measured—a metal-specific finding distinct from the Pb-centered evidence elsewhere in this table [94,95].
15 metals, incl. Al, As, Cd, Cr, Co, Cu, Fe, Pb, Mn, Hg, Ni, Se, U, V, Zn (genotoxicity mapping); Mn, Cu (histone PTM); Cd, Hg + metalwork (DNA methylation)	[Review] Systematic genotoxicity mapping + [Multi-metal] 2 experimental/epidemiological epigenetic studies	A comprehensive mapping against ATSDR’s genotoxicity framework found substantial evidence linking metal exposure to genotoxic damage in both sporadic and familial ALS, though nearly 80% of possible genotoxic endpoints remain unexplored; Mn and Cu directly alter histone H3 modifications in a yeast model relevant to ALS/FTD epigenetics; DNA methylation changes are measurably associated with self-reported Cd, Hg, and metalwork exposure history in a large ALS cohort (*n* = 855) [91,92,93]

Rows highlighted in light orange represent studies shared with the other neurodegenerative diseases covered in this review. BBB = blood–brain barrier; ICP-MS = inductively coupled plasma–mass spectrometry; GWAS = genome-wide association studies; AMSTAR = assessing the methodological quality of systematic reviews; FTD = frontotemporal dementia; [Direct] = formal mixture statistics or a designed multi-metal co-exposure, [Multi-measured] = two or more metals measured/compared but analyzed individually, [Single-metal] = one metal only, [Review] = narrative review, protocol or methodological paper.

### 4.4. Multiple Sclerosis

Unlike AD, PD, and ALS, where several of the studies reviewed above [8,21,25] explicitly modeled metal–metal interactions using WQS or BKMR, the MS literature reviewed here consists almost entirely of single-metal, one-at-a-time associations, even in studies that measured several metals from the same blood or tissue sample. None of the MS studies identified in this review applied a joint mixture-statistics model comparable to those used in the other three diseases.

A recent small-cohort study investigated Cd, As, and Pb alongside metallothionein expression in MS patients, one of the few studies considering an essential metal-handling protein jointly with multiple toxic metals in this disease. The results report that metallothionein has an important role in MS, with unexpectedly lower, not higher, values for Cd, As and Pb in MS patients compared to controls [100]. Cross-sectional studies from Iran and elsewhere have reported associations between blood or serum concentrations of multiple metals and MS clinical course or prevalence, though findings are mixed: in a Polish cohort of 151 patients, Cd and Pb levels did not differentiate functional status or disease course, with only a non-significant trend toward worse functional status at higher blood Pb [101], whereas studies in Iranian cohorts have reported significant metal–clinical associations [102,103]. Geo-environmental and soil-based studies from three independent regions have linked heavy metal burden to MS distribution, though implicating different metals in each setting. In Isfahan, Iran, proximity to steel-mill dust deposition correlated with an 18-fold higher lead concentration in the nails of MS patients compared to healthy controls [104], while a separate soil-sampling study in the same province found that absorbable Pb and Cd levels were independently associated with MS prevalence across townships, in opposing directions—Cd is reported to be positively associated and Pb negatively associated with MS prevalence [105]. In Sardinia, a population-based analysis using mixed-effects modeling identified Cu, rather than Pb or Cd, as the strongest predictor, with each 50 ppm increase in soil Cu associated with nearly three-fold higher MS odds [106]. In Finland, a toxicokinetic model linked MS clustering to acid sulphate soils that release Fe, Al, Mn, Cu, and Cd, proposing a distinct geochemical mechanism entirely [107].

Beyond these regional patterns, other studies have addressed (i) gut microbiota-heavy metal interplay—a stool metagenomic study found elevated As, Ni, Mn, and Zn but reduced Fe, Pb, titanium (Ti), and tin (Sn) in MS patients, alongside an altered gut microbial community, suggesting that gut microbiota, a modifiable target through probiotics or dietary intervention, should be evaluated alongside metal exposure itself when investigating MS pathogenesis and potential therapeutic strategies [108]; (ii) vitamin C/metal-ion imbalance distinguishing MS subtypes—despite similar disability levels and comparable neurodegenerative/neuroinflammation markers, primary progressive MS was distinguished by lower central nervous system antioxidant capacity, altered intrathecal ascorbate retention and imbalance in Mn and Cu [109]; and (iii) blood–brain-barrier leakage—As and Cd blood levels have been shown to be elevated together with S100B, the blood–brain-barrier disruption marker, with As proving the strongest correlation (63%) to S100B levels; these results suggest the two metals could possibly contribute to MS pathogenesis partly through BBB compromise [110]. This last finding is further supported at the single-neuron level, with individual human locus coeruleus neurons showing substantial variation in Se, Hg, rubidium (Rb), Fe, Cu, and Pb concentrations, offering a plausible structural link given this nucleus’s role in maintaining BBB integrity, though the small sample size warrants caution [48].

A systematic review and meta-analysis of toxic heavy metal concentrations in MS patients provides the most comprehensive synthesis available, though, consistent with the pattern above, it aggregates single-metal associations rather than characterizing mixture interactions directly [111]. This near-absence of designed mixture studies is not evidence that metal–metal interactions are less relevant to MS, but rather that the specific analytic approach central to this review has simply not yet been applied here to the same extent as in the other three diseases.

Synergism, antagonism or additivity between metals are not reported in the studies reviewed in this section. The evidence here is at most joint association in nature, or, in several cases, contradictory [100,101,111].

Table 4 summarizes the studies included in this review addressing metal exposure in relation to MS.

**Table 4:** Synthesis of studies on metal exposure and multiple sclerosis.

Metals/Mixture Studied	Design/Method	Key Findings (Synthesized)
Pb, Hg, V, Cr (review); Hg, Ag, Bi (spinal interneurons)	[Review] 1 narrative review (shared with AD/PD/ALS tables) + 1 histological aging study (shared with ALS table, non-MS tissue)	The spinal interneuron study used general-population tissue, not MS patients specifically, and is cited here only for relevance to the multifocal CNS pathology pattern proposed to underlie MS as well as ALS [62,97]
Hg, Se, Fe, Cu, Pb, Rb	[Multi-measured] Synchrotron X-ray fluorescence, single-neuron resolution, locus coeruleus tissue from 7 actual MS donors	This study used real MS-patient tissue. Individual neurons varied substantially in toxic and essential metal content, with Hg confined to a scattered neuronal subset—proposed as a structural mechanism for the non-random, multifocal destruction pattern characteristic of MS [48]
As, Cd (GSTM1 study); Cd, Pb (blood + smoking); As, Cd, Pb (DNA methylation)	[Multi-measured] 3 studies from the same research group, same Tehran cohort (~69 RRMS patients/74 controls), examining genetic polymorphism, blood levels, and epigenetics	As and Cd consistently elevated in MS patients across all three analyses. Pb showed no significant MS-vs-control difference in two of the three (only a sex difference within patients). Cd susceptibility was linked to GSTM1-null genotype and smoking; As-induced hypomethylation of ACKR3 was proposed as a specific epigenetic mechanism [102,103,112].
As, Pb, Cd	[Multi-measured] Cross-sectional, Tehran, blood metals + serum S100B (BBB-disruption marker)	MS patients showed elevated blood As and Cd alongside higher serum S100B, with As showing the strongest correlation (63%) to S100B—proposing metal-induced BBB compromise as a contributing mechanism [110]
Cd, Pb (Poland); As, Pb, Hg, Cd (meta-analysis, 16 studies); As, Cd, Pb (Turkey)	[Multi-measured] Cross-sectional cohort (*n* = 151) + systematic review/meta-analysis (*n* = 1650) + case-control (*n* = 50)	*Directly conflicting results*. The meta-analysis (largest evidence base) found Pb, As, and Cd to be significantly elevated in MS patients (Hg not significant). A Polish cohort found Cd and Pb did not differentiate functional status or disease course (only a non-significant trend at higher Pb). A Turkish case-control found the opposite direction: As, Cd, and Pb were significantly higher in controls than in MS patients. *This direct contradiction is a notable feature of the MS literature not seen to the same degree in the other three diseases* [100,101,111].
Pb, Hg (+ organic solvents)	[Multi-measured] Case-control, *n* = 217/496, gene-environment (SNP) interaction analysis	Self-reported Pb (OR = 2.03) and Hg (OR = 2.06) exposure were both significantly associated with MS; potential interactions with SNPs in TNF-a, TNF-b, VDR, MBP, and APOE were noted but flagged as requiring cautious interpretation given limited sample size [113]
Co, Cr, Ni, Pb, Zn, Cu (Sardinia); Co, Pb, Cd, Cu, Zn (Isfahan soil); Pb, Cd, Ni, Co, Mn (Isfahan dust/nail); Fe, Al, Mn, Ni, Cu, Cd (Finland)	[Multi-measured] 4 independent geo-environmental/ecological studies across 3 countries (PCA + GLMM; linear regression; dust deposition + nail biomarker; national soil mapping)	*No consistent cross-regional signal*—each study implicates a different primary metal. Cu was the strongest MS risk factor in Sardinia (OR per 50 ppm = 2.83). Pb and Cd in Isfahan soil showed opposing directions (Pb positively, Cd negatively associated with prevalence). A separate Isfahan study found nail lead to be 18-fold higher in MS patients near steel mills. A Finnish study proposed Fe, Al, and Mn leaching from acid sulphate soils as the geochemical driver of regional MS clustering. This cross-regional heterogeneity is difficult to interpret without individual-level mixture data [104,105,106,107].
As, Ni, Mn, Zn (elevated); Fe, Pb, Ti, Sn (reduced)	[Multi-measured] Stool ICP-MS + 16S rRNA gut microbiome metagenomics	MS patients showed a mixed-direction metal signature in stool—some toxic/essential metals were elevated, while others were reduced—alongside an altered gut microbial community, suggesting gut microbiota (a modifiable target via probiotics/diet) should be evaluated alongside metal exposure itself [108]
Mg, Mn, Cu, Fe, Pb, Zn, Ca	[Multi-measured] NMR + biomarker analysis, paired CSF and serum, PPMS vs. SPMS vs. controls	Faster PPMS progression was associated with diminished CNS antioxidative capacity, altered ascorbate retention, and Mg/Cu imbalance; this is among the few MS studies quantifying both toxic and essential metals in the same compartment (descriptive rather than an interaction model) [109]

Rows highlighted in light orange represent studies shared with the other neurodegenerative diseases covered in this review. CNS = central nervous system; BBB = blood–brain barrier; ICP-MS = inductively coupled plasma–mass spectrometry; GWAS = genome-wide association studies; FTD = frontotemporal dementia; NMR = nuclear magnetic resonance; PPMS = primary progressive multiple sclerosis; SPMS = secondary progressive multiple sclerosis; [Direct] = formal mixture statistics or a designed multi-metal co-exposure, [Multi-measured] = two or more metals measured/compared but analyzed individually, [Single-metal] = one metal only, [Review] = narrative review, protocol or methodological paper.

## 5. Cross-Pathology Comparison

Comparing the evidence in all four diseases discussed above reveals both convergent and disease-specific patterns. Pb, Mn, and Cu recur across all four diseases, consistent with their shared reliance on the transporter-competition and mitochondrial mechanisms, with DMT1-mediated uptake providing a plausible common entry route. As and Cd appear more prominently in both Alzheimer’s and multiple sclerosis literature, though in MS this concentration partly reflects repeated analysis of the same Iranian cohort rather than independent replication. They are often paired with Pb in three-metal designs, while Mn-V combinations are distinctive to the PD literature, reflecting shared occupational sources like welding. ALS stands out for the composition of its strongest mixture study, where the individual significant metals (Cu, Se and Zn) are essential rather than classically toxic elements. This study is independent of genetic risk, suggesting environmental mixture burden and inherited susceptibility may operate through partially separate pathways in this disease.

Disease-specific mechanistic emphasis also differs: AD evidence most directly implicates mitochondrial Cu mishandling (Pb-COX17) and antioxidant-buffering interactions (Se-Cd, Se-Pb); PD evidence most directly implicates a genetically validated kinase pathway (Mn-LRRK2) alongside direct alpha-synuclein binding; ALS evidence is strongest at the population-mixture level but comparatively thin mechanistically, with its clearest mechanistic finding (TDP-43 induction) not yet linked to a specific metal combination; and MS evidence remains at the single-metal association stage without an equivalent mechanistic anchor. This unevenness is itself informative: the gap is concentrated in MS, not randomly distributed.

Table 5 synthesizes and compares the four disease-specific profiles across seven evidence dimensions, highlighting where mixture evidence is strong, contested, or entirely absent.

**Table 5:** Cross-disease comparison of metal-mixture evidence in AD, PD, ALS and MS.

	AD	PD	ALS	MS
**Best-supported mixture**	Al-Pb-Li (synergistic); Cu-Pb-Se (antagonistic)	Mn-V (synergistic, 1 designed 4-arm study)	Multi-metal risk score (Cu-Se-Zn individually significant)	None
**Formal mixture-statistics evidence**	Strong—6 cohorts (WQS/BKMR/qgcomp)	Moderate—2 cohorts (BKMR/WQS/QGC)	Strong but singular—1 cohort using a composite environmental risk score	None
**Experimental co-exposure studies**	Yes—4 models (organotypic slices, APP/PS1 mice, 2 rat models)	Yes—1 confirmed (Mn-V, 4-arm design)	None with proper multi-arm design	None
**Tested synergy/antagonism**	1 synergy; antagonism best represented (Se vs. Cu-Pb, As-Cd-Pb, Cd)	2 synergies (Mn-V; Mn-Cr)	None tested—joint association only; 1 null result (Pb-Cd-Hg, CSF)	None
**Essential-metal modifiers**	Se (strong); Zn	Zn (protective); Se (deficiency = risk)	Cu, Se, Zn—elevated, not deficient (opposite direction from AD/PD pattern)	Not systematically tested
**Main mechanistic pathway(s)**	Mitochondrial Cu mishandling (COX17); autophagy–lysosomal impairment; DNA methylation convergence	LRRK2 activation (Mn, redox-dependent); direct α-synuclein binding	TDP-43 disruption (Pb, MeHg)	BBB disruption (As-Cd/S100B); gut–metal interplay
**Overall consistency of evidence**	Moderate–high	Moderate—Mn/Pb recur, but literature notes “some but not all” studies present this correlation	Mixed—strongest population signal of all four, but CSF-level data contradictory	Low—directly conflicting results across cohorts
**Key research gap**	Interaction surfaces rarely reported even when BKMR/WQS used	Broader mixture-interaction testing in PD is still needed	No designed experimental mixture studies; no formal interaction testing despite strong epidemiological signal	Complete absence of mixture-statistics/designed co-exposure approaches

Moderate = replicated in 2 cohorts, but inconsistent in older literature; moderate–high = replicated across large cohorts; mixed = strong signal, but CSF data null; low = small studies, no replication, no formal mixture stats. BBB = blood-brain barrier.

## 6. Methodological Considerations for Mixture Research

The mixture-statistics methods underlying the strongest studies reviewed, weighted quantile sum (WQS) regression and Bayesian kernel machine regression (BKMR), carry specific limitations. WQS assumes all mixture components act in the same direction, an assumption that is violated whenever an essential metal (Se, Zn) is included alongside toxic metals [114]. BKMR relaxes this assumption and models non-linear, non-monotonic joint effects, including genuine interaction terms, but requires larger samples and is more computationally demanding [115]; several of the strongest studies reviewed, including the MESA cohort [21] and welder neuroimaging studies, used BKMR for this reason [31,32].

A second limitation concerns exposure matrix: the postmortem brain-versus-ventricular-fluid comparison in AD [33] suggests that fluid-based measurements do not necessarily represent brain tissue concentrations, with implications for nearly all epidemiological studies reviewed here that rely on blood or urine as an accessibility-driven proxy. A third limitation is temporal: urinary and blood metal concentrations largely reflect recent exposure, while neurodegenerative pathology accumulates over years to decades, creating a potential mismatch between the exposure window captured and the etiologically relevant period. Hair and nails integrate longer periods of time and could be more useful, but they risk external contamination. Cross-study comparison should be treated with caution since these matrices are not interchangeable.

A further limitation concerns confounding. Factors such as smoking, occupational exposure, diet, socioeconomic status, renal function, geographic location, sex, age and co-exposure to non-metal toxicants likely contribute to the heterogeneity observed across studies. The covariate adjustment was inconsistent throughout the literature review: some studies controlled for smoking and age, others for diet, and others for genetic risk alone.

A methodological strength worth highlighting is the sequential, multi-method approach used in the welder neuroimaging studies [31]: dose–response patterns were first explored via loess plots, then tested with linear and polynomial regression, before applying BKMR as the final step to capture non-linear mixture and interaction effects. This layered approach, rather than relying on BKMR alone, allows internal cross-validation of findings across methods with different assumptions, and could serve as a template for future mixture studies in Alzheimer’s, Parkinson’s, ALS, and MS alike.

## 7. Translational Biomarkers

Biomarkers connecting metal-mixture exposure offer a translational bridge to mitochondrial dysfunction. Mitochondrial DNA copy number in peripheral blood has been associated with As exposure specifically [116] and with multiple-metal mixture exposure in a large Chinese cohort [117], and increased newborn mitochondrial DNA copy number has been linked to prenatal As metabolite exposure in a birth control study [118]—extending the principle that metal-induced mitochondrial perturbation is detectable as a peripheral signal, though mtDNA copy number is nonspecific and should be interpreted cautiously alone.

The literature describes a well-established biomarker category for metal mixtures specifically: oxidative stress effect biomarkers, particularly 8-hydroxy-2′-deoxyguanosine (8-OHdG), malondialdehyde, and the GSSG/GSH (oxidized glutathione/reduced glutathione) ratio, have been validated against formally modeled metal mixtures (WQS/BKMR) in several independent populations, from adolescents to occupationally exposed adults [119,120]. Within this review’s own evidence base, 8-OHdG mediated approximately 11.6% of the combined Mn-Pb effect on PD risk [73], emphasizing this biomarker’s applicability to metal-mixture neurotoxicity in the diseases under review. While the mixture-statistics/biomarker combination is already established for oxidative stress, the mitochondrial-dysfunction biomarker category has not yet been paired with a formal metal-mixture design in any of the diseases reviewed here.

## 8. Perspectives and Future Directions

The genuine gap identified above points toward two candidates worth examining more thoroughly: circulating GDF15 and FGF21, more recently validated mitochondrial dysfunction biomarkers, established in primary mitochondrial disease [121], pediatric mitochondrial disorders [122], and insulin resistance/PCOS [123]. Neither has been validated specifically for metal-mixture exposure across the four diseases reviewed, representing a concrete near-term opportunity: combining an established mitochondrial biomarker (GDF15/FGF21) with an established mixture-exposure design (WQS/BKMR) has not, to our knowledge, been attempted in any of the four diseases.

Multiple sclerosis research should adopt the designed mixture-statistics approaches already applied to the other three diseases; existing MS cohorts with multi-metal measurements could often be reanalyzed without new sample collection. The organotypic brain slice and transgenic-mouse paradigms used for AD [28] and PD [71] suggest that designed co-exposure experiments in disease-relevant models are feasible; extending these to ALS and MS models, with essential-metal co-treatment arms, would directly test the essential–toxic modulator mechanism under controlled conditions. Given the tissue–fluid discrepancy documented in AD and the temporal mismatch discussed in this review, future studies would benefit from routinely reporting exposure matrix and combining short-term (blood, urine) with longer-term (bone, hair, nail) biomarkers, an approach so far applied only inconsistently.

## 9. Conclusions

This review compared, across four major neurodegenerative diseases, the evidence for cumulative and interactive effects of heavy metal mixtures. First, the general mechanism across all four diseases were described (transporter-level competition, chemical sequestration/antioxidant buffering, and directly measured synergism or antagonism), though specific metal pairs and evidence depth differ substantially. Second, mixture evidence strength is highly uneven: AD and PD each have multiple designed co-exposure experiments with disease-specific mechanistic anchors; ALS has the single strongest population-level mixture association identified (multi-metal exposure, independent of genetic risk), but a comparatively thin mechanistic literature; and MS has almost no designed mixture studies despite considerable single-metal data, treated here explicitly as a research gap rather than a negative finding about MS biology. Third, essential metals like Se, Zn, Cu, and Fe are an active modulator of toxic-metal effects wherever designed interaction studies exist. Future mixture research in all four diseases, and in multiple sclerosis specifically, would be strengthened by treating essential-metal status as a designed covariate and by adopting the mixture-statistics and experimental co-exposure methods already validated in the Alzheimer’s and Parkinson’s disease literature reviewed here.

## Data Availability

No new data were created or analyzed in this study.

## Abbreviations

8-OHdG	8-Hydroxy-2′-deoxyguanosine
6-OHDA	6-Hydroxidopamine
Aβ	Amyloid-beta
AD	Alzheimer’s Disease
Ag	Silver
Al	Aluminum
ALS	Amyotrophic Lateral Sclerosis
AMSTAR	Assessing the Methodological Quality of Systematic Reviews
APP	Amyloid Precursor Protein
As	Arsenic
Au	Gold
Ba	Barium
BBB	Blood–Brain Barrier
Bi	Bismuth
BKMR	Bayesian Kernel Machine Regression
Ca	Calcium
Cd	Cadmium
CNS	Central Nervous System
Co	Cobalt
Cr	Chromium
Cs	Cesium
CSF	Cerebrospinal Fluid
Cu	Copper
DMT-1	Divalent Metal Transporter 1
Fe	Iron
FPN	Ferroportin
FTD	Frontotemporal Dementia
GSSG/GSH	Oxidized Glutathione/Reduced Glutathione Ratio
GWAS	Genome-Wide Association Studies
Hg	Mercury
ICP-MS	Inductively Coupled Plasma–Mass Spectrometry
LC50	Lethal Concentration 50
Li	Lithium
LRRK2	Leucine-Rich Repeat Kinase 2
MCI	Mild Cognitive Impairment
MESA	Multi-Ethnic Study of Atherosclerosis
MeHg	Methylmercury
Mg	Magnesium
Mn	Manganese
Mo	Molybdenum
MPTP	1-Methyl-4-phenyl-1,2,3,6-tetrahydropyridine
MRI	Magnetic Resonance Imaging
MS	Multiple Sclerosis
NHANES	National Health and Nutrition Examination Survey
Ni	Nickel
NMR	Nuclear Magnetic Resonance
PD	Parkinson’s Disease
Pb	Lead
Pd	Palladium
PPMS	Primary Progressive Multiple Sclerosis
Rb	Rubidium
ROS	Reactive Oxygen Species
Sb	Antimony
Se	Selenium
Sn	Tin
SPMS	Secondary Progressive Multiple Sclerosis
Tl	Thallium
Ti	Titanium
U	Uranium
V	Vanadium
W	Tungsten
WQS	Weighted Quantile Sum Regression
Zn	Zinc
